# United Confederate Veterans’ Reunion at Louisville

**Published:** 1900-03

**Authors:** Preston B. Scott

**Affiliations:** Chairman Medical Committee; Atlanta Journal-Record of Medicine, Atlanta, Ga.


### CORRESPONDENCE.

UNITED CONFEDERATE VETERANS,
REUNION OF 1900—MAY 30, 31, JUNE 1, 2, 3.
LOUISVILLE, KY., February 17, 1900.

*Dear Doctor:*

The Medical Committee desires to make a feature of the Louisville Reunion the assembling and entertainment of physicians who were surgeons of the Army and Navy of the Confederate States, as well as those who are veterans and sons of veterans.

Let me ask them through the favor of your columns to send me their names, so that the committee may communicate directly with them.

Very respectfully,

(Dictated.)

PRESTON B. SCOTT,
Chairman Medical Committee.

DR. L. B. GRANDY,
Editor ATLANTA JOURNAL-RECORD OF MEDICINE, Atlanta, Ga.

---

### A NEW PROCEDURE IN THE TREATMENT OF FOREIGN BODIES IN THE NASAL FOSSÆ OF CHILDREN.

Feleizet (*Jour. de Clin. et de Therap. Infantiles,* Vol. VI., No. 48): Thirty-one cases of foreign bodies in the nose were seen among children ranging from four weeks to twelve years of age. The procedure, which gave excellent results in every case, consisted in forcing out the obstruction by means of a syringe of warm water applied to the opposite nostril. A syringe of a capacity of from three to five hundred grams is used, and the injection is begun slowly and carefully in order that the Eustachian tube shall have time to close and the palate to contract. Then the force of the stream is increased, and resistance is encountered and overcome; at the same time the foreign body is either forced out of the nostril, or moved to within easy grasp by the forceps. No accident to the middle ear has ever resulted. If the diagnosis be obscure this maneuver will readily prove the presence or absence of an obstruction, and that without the pain and danger of instrumental exploration of the nasal fossæ in young children.—*Arch. Ped.*

